# Online Reference Template Adaptation for Robust LiDAR-Based Pose Tracking of Non-Cooperative Targets

**DOI:** 10.3390/s26217033

**Published:** 2026-11-06

**Authors:** Fengxiang Wang, Genghua Huang, Xiangfeng Liu, Rong Shu

**Affiliations:** 1 College of Surveying and Geo-Informatics, Tongji University, Siping Road, Shanghai 200092, China; 2280235@mail.tongji.edu.cn; 2 Key Laboratory of Space Active Opto-Electronic Technology, Shanghai Institute of Technical Physics, Chinese Academy of Sciences, Yu Tian Road, Shanghai 200083, China; genghuah@mail.sitp.ac.cn (G.H.); xiangfeng_liu@163.com (X.L.); 3 University of Chinese Academy of Sciences, Beijing 100049, China; 4 Shanghai Research Center for Quantum Sciences, Shanghai 201315, China; 5 Shanghai Branch, Hefei National Laboratory, Shanghai 201315, China

**Keywords:** LiDAR pose tracking, non-cooperative target, online reference template adaptation, point cloud registration

## Abstract

**Highlights:**

**What are the main findings?**

- Online adaptation reduces the mean registration RMSE by approximately 31% relative to the fixed simplified CAD template and avoids tracking interruption during target rotation.
- Online adaptation maintains stable roll estimates as the visible surface shrinks to a locally planar region, whereas the fixed CAD template drifts by more than 10°.

**What are the implications of the main findings?**

- Selected observations improve template agreement without multi-view reconstruction.
- Small residuals can coexist with attitude drift when visible geometry is limited.

**Abstract:**

Discrepancies between geometric priors and LiDAR observations can limit pose tracking of non-cooperative targets. This paper proposes online reference template adaptation that incorporates observations selected through registration-quality, incident-angle, and temporal-support criteria into a CAD-derived template. Two numerical simulations with known pose ground truth and two ground-based experiments evaluate the method under target rotation and close-range approach. In the approaching simulation, online adaptation reduces translation and rotation RMS errors from 1.44 to 0.52 cm and from 1.10° to 0.40°, respectively. In the rotating simulation, the corresponding errors decrease from 4.61 to 4.37 cm and from 3.97° to 3.32°. In the ground-based rotational experiment, online adaptation achieves a mean registration RMSE of 3.29cm, approximately 31% lower than the fixed CAD baseline and close to the offline reconstructed baseline (3.05cm), while reducing threshold exceedances from 32 to four isolated frames. In the close-range experiment, roll estimates remain comparatively stable as the visible region shrinks, whereas the fixed CAD baseline exhibits more than 10° of roll drift. These results support improved pose accuracy in the tested simulations and greater tracking stability in the ground-based experiments, without dedicated prior multi-view reconstruction.

## 1. Introduction

Accurate relative pose estimation of non-cooperative targets is a fundamental capability for autonomous rendezvous, proximity operations [1], on-orbit servicing [2], and active debris removal [3]. Compared with passive imaging sensors, light detection and ranging (LiDAR) directly provides three-dimensional geometric measurements and is less dependent on ambient illumination, making it attractive for close-range relative navigation [4].

Depending on the availability of prior geometric information, pose estimation methods for non-cooperative targets can be generally divided into model-based approaches and reconstruction-based approaches [5]. For known targets with available prior information, such as target shape, size, or three-dimensional models, model-based methods estimate the target pose by registering sensor observations with the predefined target model [6,7]. Among these approaches, LiDAR-based model registration methods have been widely investigated for close-proximity pose estimation because they directly exploit three-dimensional geometric constraints. Learning-based methods provide another route to pose estimation: LSPnet directly regresses spacecraft poses from monocular images without requiring an explicit three-dimensional model at inference [8]. In other application domains, RGB-D measurements have also been used for real-time, full-range six-degree-of-freedom markerless head pose estimation [9].

**Figure 1:**
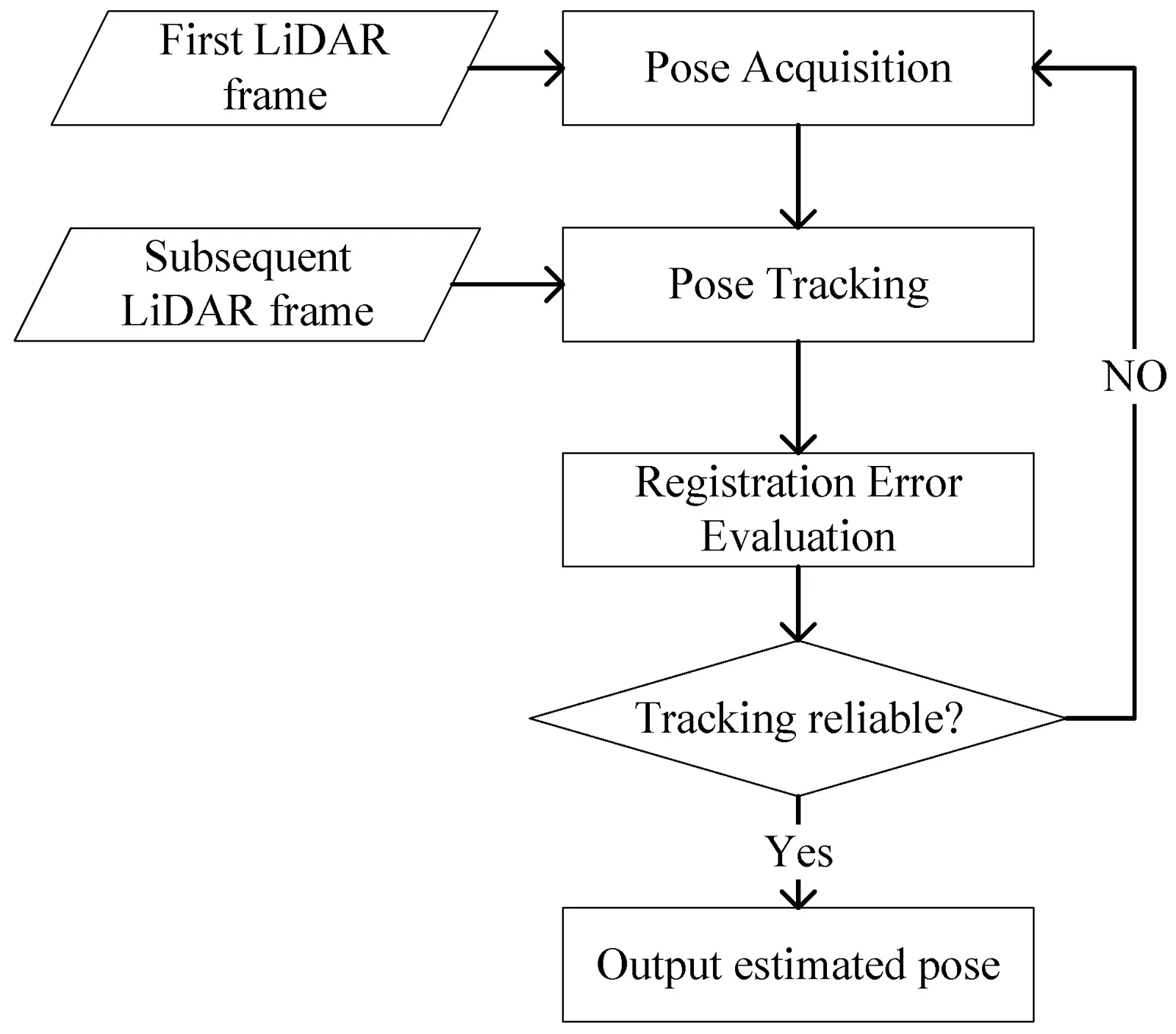
Acquisition and tracking in model-based pose determination.

In model-based pose estimation, the estimation process generally operates through two successive stages, namely pose acquisition and pose tracking, as illustrated in Figure 1 [10]. Pose acquisition is performed when the first observation becomes available and no reliable prior pose estimate exists. Existing acquisition methods therefore solve a global registration problem under large initial pose uncertainty. Representative approaches include 3D template matching methods based on predefined target models [11,12] and point-cloud feature-based methods that exploit geometric descriptors for global correspondence establishment, such as polygonal aspect hashing [13] and OUR-CVFH [14]. Once an initial pose has been obtained, the system switches to pose tracking, where the target pose is continuously updated using the previous pose estimate as the initialization for local registration. In particular, the Iterative Closest Point (ICP) algorithm and its variants are widely adopted to refine the rigid transformation between the current observation and the reference model [13,15,16].

Since pose tracking relies on local registration, a tracking-quality assessment is usually used to monitor the estimated pose. Registration metrics, such as the residual, are evaluated at each frame. A residual above a predefined threshold indicates degraded tracking quality; to avoid switching modes in response to a transient single-frame fluctuation, the system returns to pose acquisition only when the exceedance persists according to the switching policy, as illustrated in Figure 1. This acquisition–tracking architecture allows recovery from tracking failure, but continuous tracking is preferable to repeated reacquisition during dynamic close-proximity operations. Moreover, a small residual measures geometric agreement and does not necessarily indicate an accurate pose when the visible geometry provides weak constraints.

The performance of model-based methods depends on the quality and suitability of the reference model. Existing studies have explored several ways to reduce discrepancies between the model and the physical target. Target-model uncertainty has been investigated in vision-based relative pose estimation [17]. Dedicated multi-view observations and three-dimensional reconstruction can provide a reference that more closely represents the measured target geometry [18,19]. Another direction uses observation-derived representations to support pose estimation without a predefined CAD model, including recent NeRF-based approaches [20,21]. Within model-based LiDAR tracking, Perfetto et al. addressed geometric discrepancies in major target components, particularly offsets between structural elements that introduce systematic pose estimation errors [22]. However, agreement in the overall target geometry does not necessarily ensure that the reference model adequately represents the surface details captured by LiDAR observations.

Related discrepancies between synthetic and real observations have also been investigated in learning-based spacecraft pose estimation. The SPEED+ dataset supports evaluation across this domain gap [23], while Liu and Yu investigated a self-supervised approach to reduce its effect on non-cooperative spacecraft pose tracking [24]. Feng et al. further investigated online test-time adaptation for spacecraft pose estimation, using a dynamic memory bank and teacher–student learning to adapt to unseen target-domain observations [25]. This learning-based adaptation complements efforts to update the geometric reference used in LiDAR registration. For LiDAR-based tracking, extreme observation conditions, such as large incident angles or a severely restricted field of view (FoV), can produce sparse and incomplete point clouds. Registering these observations to a reference model remains challenging, particularly when few distinctive geometric features are visible.

To address these challenges, this paper proposes an online reference template adaptation framework for LiDAR-based pose tracking. Whereas Perfetto et al. reconstruct triangular meshes and match extracted planes to identify and correct geometric offsets [22], our method directly adapts a CAD-derived point cloud template through reliable observation selection and incremental fusion. Observations are selected using registration-quality, incident-angle, and temporal-support criteria and incorporated into the reference template during tracking. The method retains the model-based tracking framework and aims to reduce persistent surface-detail mismatch and maintain tracking stability under degraded sensing conditions, without a dedicated prior multi-view reconstruction.

## 2. Materials and Methods

**Figure 2:**
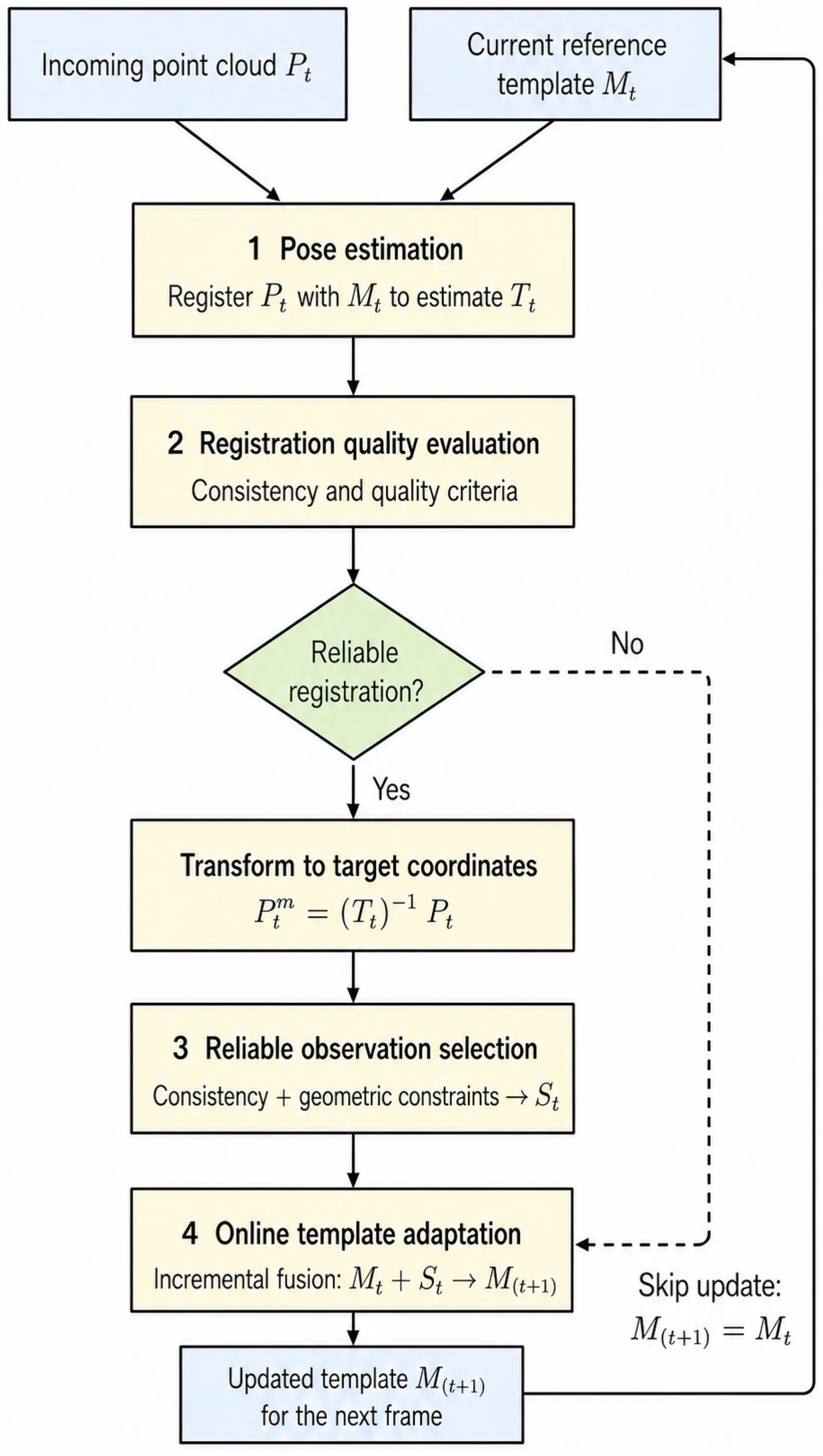
Overall workflow of the proposed method.

The proposed online reference template adaptation framework is developed within the conventional model-based pose tracking architecture introduced in Section 1. Unlike conventional methods that employ a fixed reference template throughout the entire tracking process, the proposed framework progressively adapts the reference template according to reliable LiDAR observations while preserving the original pose acquisition and pose tracking framework. The overall workflow is illustrated in Figure 2. It consists of three major modules, namely pose estimation and registration quality evaluation, reliable observation selection, and online reference template adaptation.

Let $P_{t}={\left\{p_{t,i}\right\}}_{i=1}^{N_{t}}$ denote the LiDAR point cloud at frame *t* in sensor coordinates. The reference template $M_{t}={\left\{m_{t,j}\right\}}_{j=1}^{N_{m}}$ is expressed in target coordinates. Registration between these point clouds gives the pose estimate $T_{t}=\left[R_{t},t_{t}\right]\in SE\left(3\right)$. If the registration passes the residual criterion, the observation is transformed into target coordinates,

(1) $$P_{t}^{m}=T_{t}^{-1}P_{t},$$

where $P_{t}^{m}$ denotes the current observation represented in the target coordinate system. Observations that pass registration-consistency, incident-angle, and temporal spatial-support checks are incrementally fused into the reference template for use in the next frame. If the current registration is regarded as unreliable, the reference template remains unchanged. The implementation details of the three modules are presented in the following subsections.

### 2.1. Pose Estimation and Registration Quality Evaluation

Considering the computational efficiency and implementation simplicity required for on-chip processing, a customized lightweight weighted ICP algorithm is developed for pose estimation. It follows the ICP registration procedure [26,27] and assigns scalar weights according to each point’s geometric feature class.

The 64-beam scan structure provides neighborhoods for geometric feature extraction. Following LOAM [28], local smoothness separates edge and planar points. The observation is partitioned as

(2) $$P_{t}=P_{t}^{e}\cup P_{t}^{p}\cup P_{t}^{o},$$

where $P_{t}^{e}$ and $P_{t}^{p}$ denote the edge and planar feature subsets, respectively, and $P_{t}^{o}$ contains the remaining points.

All observed points are registered against the complete template. Feature classes share the same nearest-neighbor search and residual definition, with scalar weights assigned as

(3) $$w_{t,i}=\left\{\begin{array}{cc} w_{e}, & p_{t,i}\in P_{t}^{e},\\ w_{p}, & p_{t,i}\in P_{t}^{p},\\ 1, & p_{t,i}\in P_{t}^{o}, \end{array}\right.$$

Here, $w_{e}>1$ and $0<w_{p}<1$ denote the weights assigned to edge and planar points, respectively, relative to the unit weight assigned to the remaining points. This weighting increases the relative contribution of edge points and reduces the dominance of extensive planar regions in the registration objective. When observations are incomplete but informative edges remain visible, these structural features can provide stronger relative constraints on the pose estimate. The weighting is intended to mitigate tracking degradation associated with partial observations and predominantly planar geometry.

Let $C_{t}$ be the correspondence set at an ICP iteration. The weighted registration objective is

(4) $$T_{t}=\arg\min_{T\in SE(3)}\sum_{\left(i,j\right)\in C_{t}}w_{t,i}{∥p_{t,i}-\left(Rm_{t,j}+t\right)∥}_{2}^{2},$$

where T=[R,t] maps target-template coordinates to sensor coordinates. Each observed point $p_{t,i}$ is paired with a template point $m_{t,j}$. The optimized transformation $T_{t}$ gives the pose estimate at frame *t*.

Registration RMSE, denoted by $r_{t}$, is computed as the unweighted root mean square distance between observed LiDAR points and their corresponding reference-template points after registration, using the final correspondence set $C_{t}$:

(5) $$r_{t}=\sqrt{\frac{1}{|C_{t}|}\sum_{\left(i,j\right)\in C_{t}}{∥p_{t,i}-\left(R_{t}m_{t,j}+t_{t}\right)∥}_{2}^{2}},$$

where $R_{t}$ and $t_{t}$ are the rotation matrix and translation vector contained in the estimated pose $T_{t}$. The reference is the template used by each method. Thus, $r_{t}$ measures point-to-template geometric agreement.

A frame is eligible for observation selection only if

(6) $$r_{t}<\tau_{r},$$

where $\tau_{r}$ is the registration-residual threshold. Passing this gate permits point selection and template updating; it does not independently verify the pose. The following geometric and temporal criteria provide additional screening.

### 2.2. Reliable Observation Selection

Not every observed point is suitable for template updating: directly incorporating all observations may introduce erroneous geometric structures caused by registration errors, sensor noise, or missing measurements, progressively deviating the template from the actual target geometry. Reliable observations must therefore be geometrically consistent with the current reference template, reliable with respect to the LiDAR incident angle, and supported by repeated observations over time. Recalling Equation (1), each point in $P_{t}^{m}$ is evaluated using the following criteria.

(1) Registration Consistency
  Each observed point must be close to its nearest neighbor in the template, i.e., $d_{i}={∥p_{t,i}^{m}-m_{t,j}∥}_{2}<\tau_{d}$, where $m_{t,j}$ is the nearest point in $M_{t}$.
(2) Incident-Angle Reliability
  Large incident angles tend to produce sparse measurements and less stable point localization. Therefore, observations are retained only when their incident angle satisfies $\theta_{i}<\tau_{\theta }$.
(3) Temporal Spatial Support
  Since LiDAR returns do not repeat at identical coordinates, a fixed voxel grid is initialized from the template in its coordinate system. Each registered point is assigned an integer voxel index by component-wise flooring:
  (7) $$v\left(i\right)=\left\lfloor \frac{p_{t,i}^{m}}{d_{v}}\right\rfloor$$
  For each voxel, the counter increases once when at least one point passes the distance and incident-angle criteria in a frame; otherwise it resets to zero. A point is retained when its voxel is supported in $N_{\min}$ consecutive frames.

Points passing all three criteria form the reliable observation set

(8) $$S_{t}=\left\{p_{t,i}^{m}|d_{i}<\tau_{d},\;\theta_{i}<\tau_{\theta },\;N_{v(i),t}\geq N_{\min}\right\}.$$

Together, these screening criteria also help limit the incorporation of unreliable measurements caused by sensor noise or multipath returns. The distance criterion rejects points that deviate substantially from the reference surface, while the incident-angle criterion restricts observations acquired under unfavorable viewing geometry. Temporal spatial support reduces the inclusion of intermittent outliers. However, these geometric checks do not explicitly identify multipath propagation, and persistent artifacts close to the reference surface may still pass the screening.

### 2.3. Online Template Adaptation

Since $S_{t}$ is already represented in the target coordinate system, it can be directly integrated into $M_{t}$ through an incremental update instead of template replacement. The reliable observations are first combined with the current reference template:

(9) $${\tilde{M}}_{t+1}=M_{t}\cup S_{t}.$$

Since continuously accumulating observations from consecutive frames may introduce redundant points and cause uncontrolled growth of the template size, voxel down-sampling is subsequently applied:

(10) $$M_{t+1}=V\left({\tilde{M}}_{t+1},d_{v}\right),$$

where V(·) denotes the voxel down-sampling operator with voxel resolution $d_{v}$. This removes spatially redundant observations and limits template growth. The updated template $M_{t+1}$ is then used as the reference for pose estimation in the following frame.

Template maintenance involves candidate voxel accumulation, temporal-support checking, and template fusion. Let $n_{t}$ denote the number of candidate observation points, $V_{t}$ the number of stored candidate voxels, and $s_{t}$ the number of accepted points to be fused. Updating the per-voxel statistics requires $O(n_{t})$ operations once voxel associations are established, while checking the stored candidates requires $O(V_{t})$ operations. For a sorting-based implementation, voxel association and ordering introduce approximately $O(\left(n_{t}+V_{t}\right)\log\left(n_{t}+V_{t}\right))$ work, and fusion with voxel downsampling requires approximately $O(\left(N_{m}+s_{t}\right)\log\left(N_{m}+s_{t}\right))$ work. The persistent storage requirement is $O(V_{t}+N_{m})$. Thus, maintenance cost depends on the occupied candidate voxels and template size; growth of the candidate history can increase both runtime and memory consumption during prolonged tracking.

### 2.4. Sensor Specifications and Algorithm Configuration

Table 1 consolidates the LiDAR sensor specifications and baseline ICP and template-adaptation parameters used as the configuration reference for the simulations and ground-based experiments. The sensor employs a 1×64 linear laser array with reciprocating and stepped scanning. Each emission covers approximately 20°×2.5°, and the full angular field of view is 20°×20°. Both the simulations and the ground-based experiments use this configuration.

All algorithms were implemented in MATLAB R2026a and executed offline on a laptop equipped with an AMD Ryzen AI 9 HX 370 processor and 32 GB of RAM, running 64-bit Windows 11.

## 3. Simulation Experiments

### 3.1. Simulation Setup

Two simulated LiDAR scenarios were evaluated to quantify pose-estimation performance against known ground truth. Simulated observations were generated from a highly detailed target mesh incorporating a covering layer, which comprised 12,982,876 triangular faces, whereas the reference template was sampled from a coarser CAD mesh of 4,354,151 faces. The two models shared the same overall geometry and differed primarily in the representation of surface details; this setup therefore examined how surface-detail mismatch between the reference model and the observations affected pose estimation. The sensor and algorithm configuration followed Section 2.4. Within this configuration, the fixed-template method retained the initial reference throughout each sequence, whereas the adaptive method progressively updated the template with observations that passed the screening criteria.

In addition to registration RMSE, performance was evaluated using translation and rotation errors relative to the simulation ground truth. Let $t_{t}$ and $R_{t}$ denote the estimated translation and rotation, and let $t_{t}^{\mathrm{gt}}$ and $R_{t}^{\mathrm{gt}}$ denote their ground-truth counterparts, using the same target-to-sensor transformation convention. The translation error was defined as

(11) $$e_{\mathrm{trans},t}={∥t_{t}-t_{t}^{\mathrm{gt}}∥}_{2}$$

and the rotation error was defined as the geodesic angle between the estimated and true rotations:

(12) $$e_{\mathrm{rot},t}=\arccos\;\left(\frac{\mathrm{tr}\;\left[R_{t}{\left(R_{t}^{\mathrm{gt}}\right)}^{T}\right]-1}{2}\right).$$

### 3.2. Approaching Scenario

#### 3.2.1. Scenario Setup

The sequence comprised 71 frames. The target approached along the sensor-frame X axis from 10 to 3 m, with a displacement of 0.1 m between consecutive frames. Translation along Y and Z, as well as roll, pitch, and yaw, remained zero. This constant-attitude approach was used to evaluate pose-estimation stability as the observation conditions changed.

#### 3.2.2. Results and Discussion

**Figure 3:**
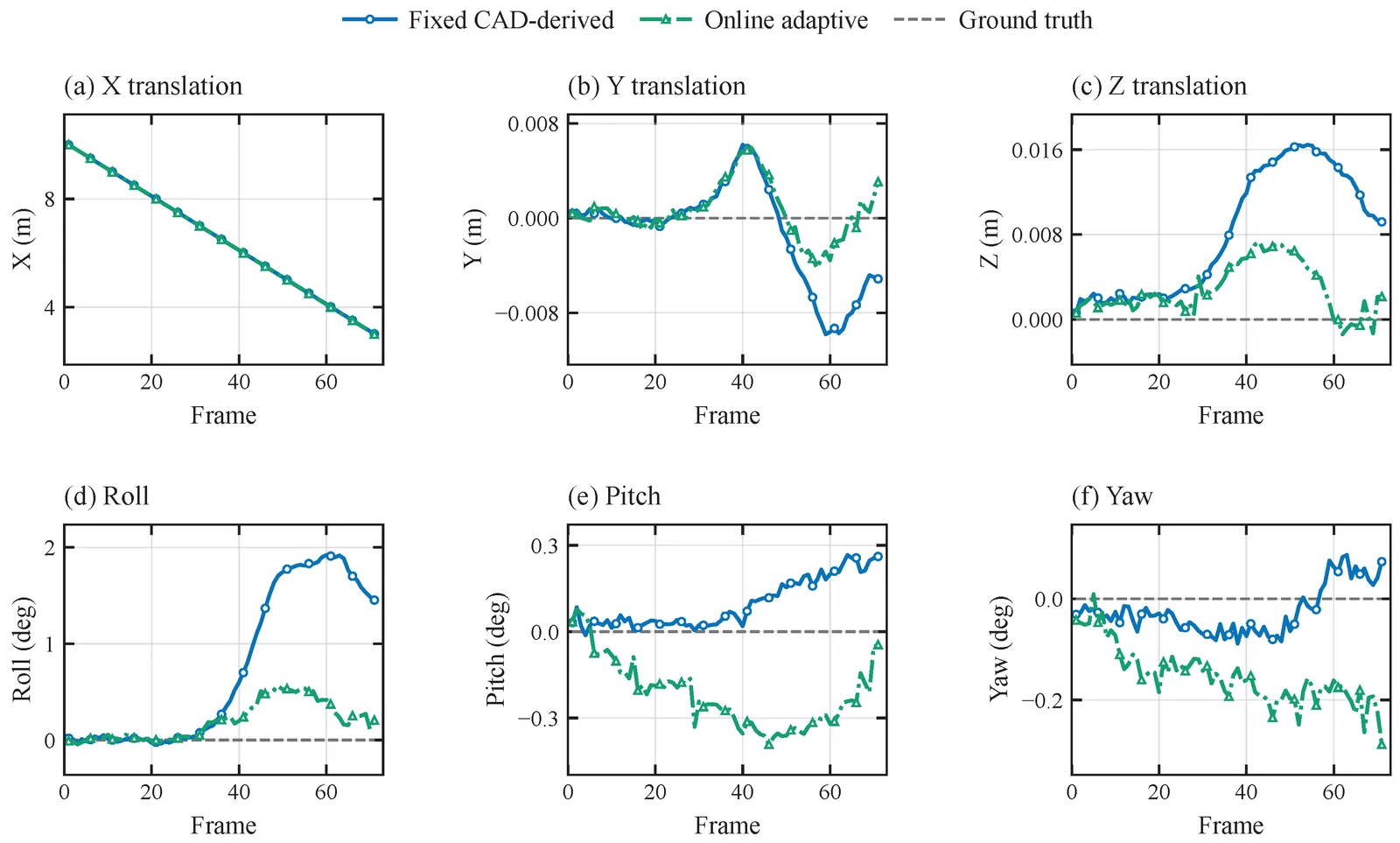
Pose trajectories during the simulated approach: (**a**–**c**) translations along X, Y, and Z; (**d**–**f**) roll, pitch, and yaw. Gray dashed lines indicate ground truth.

Figure 3 compares the estimated three-axis translations and attitudes with ground truth. Both methods followed the approach along X, while differences were more apparent in the transverse translations and attitude components. The fixed-template roll estimate progressively departed from the zero-degree ground truth in the latter part of the sequence, reaching a maximum absolute deviation of 1.92°, compared with 0.56° for the adaptive method. Online adaptation reduced the sequence-wide translation RMS error from 1.44 to 0.52 cm and the rotation RMS error from 1.10° to 0.40°. The translation improvement was most evident in the X and Z components during the latter part of the approach, while the reduction in rotation error was mainly attributable to the smaller roll deviation.

The late-sequence roll drift is consistent with weakening geometric constraints as the target approaches. With a fixed angular field of view, the surface area covered by the LiDAR decreases at shorter ranges, and the visible geometry becomes predominantly planar. Rotation about the approximately X-directed surface normal, corresponding to roll in this configuration, is then weakly constrained by the smooth CAD reference. Surface details can provide additional geometric anchors for this rotation. By incorporating selected observations, the online template captures surface details absent from the initial CAD reference and provides additional constraints on roll, consistent with the smaller deviation observed in this sequence.

### 3.3. Rotating Scenario

#### 3.3.1. Scenario Setup

In the second simulation, the LiDAR remained stationary and the target origin was fixed at ${\left[30,0,0\right]}^{T}$ m in sensor coordinates. The target rotated about its own Z axis at 5°/s, with zero roll and pitch. A total of 70 frames were sampled at 1 Hz, covering t=0–69 s and an accumulated yaw rotation of 0–345°. This scenario introduced changing views of the target at a constant range, complementing the shrinking field-of-view coverage in the approaching scenario.

#### 3.3.2. Results and Discussion

**Figure 4:**
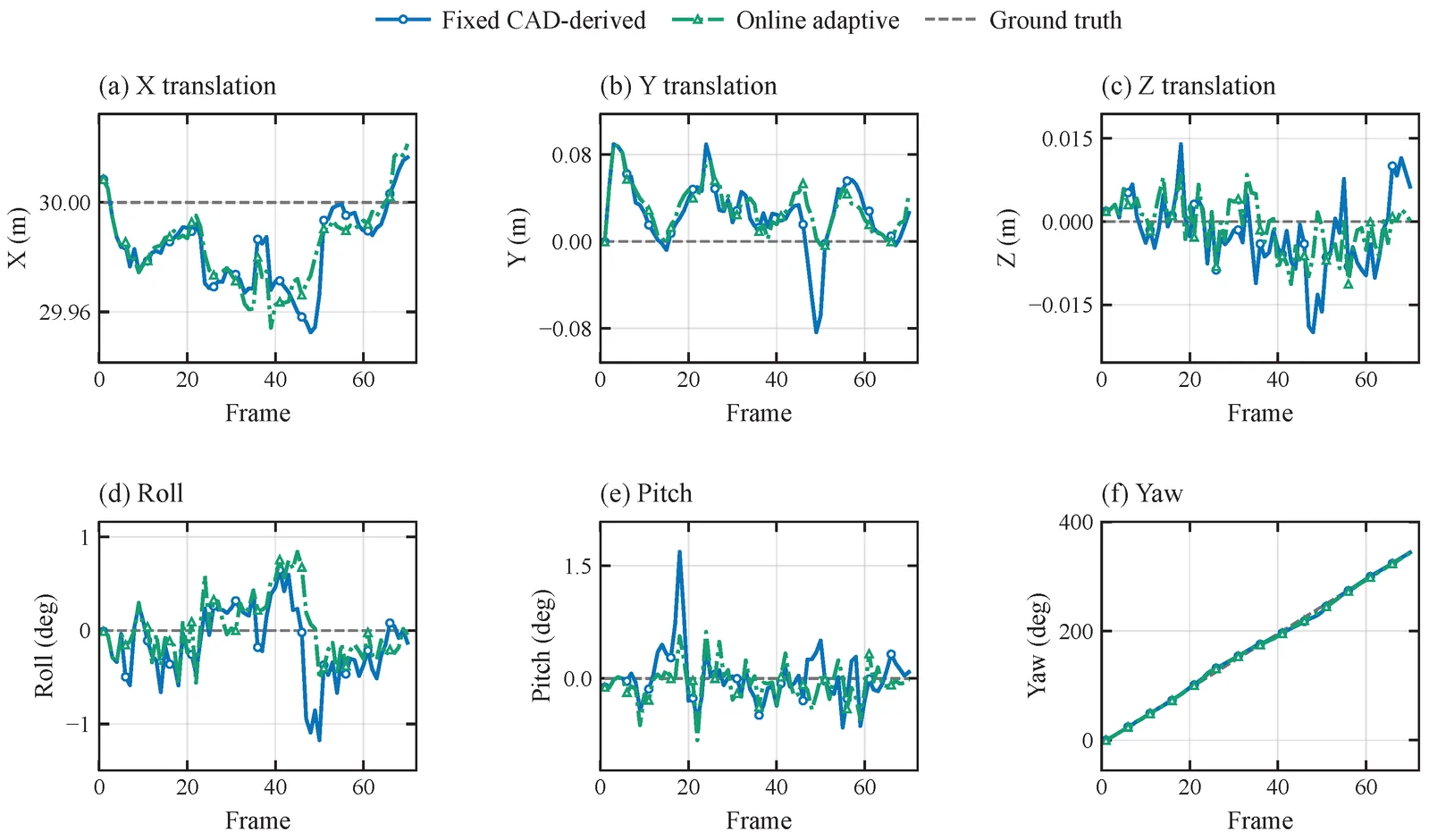
Pose trajectories during the simulated rotation: (**a**–**c**) translations along X, Y, and Z; (**d**–**f**) roll, pitch, and yaw. Gray dashed lines indicate ground truth. Yaw angles are continuously unwrapped to show the full 0–345° rotation.

Figure 4 compares the estimated translations and attitudes with the known rotating trajectory. Both methods followed the overall yaw rotation, although local departures from ground truth remained. The maximum absolute roll and pitch deviations were 1.17° and 1.69° for the fixed template, compared with 0.84° and 0.82° for the adaptive template.

Online adaptation reduced the sequence-wide translation RMS error from 4.61 to 4.37 cm and the rotation RMS error from 3.97° to 3.32°. In the large-incidence-angle portions of the sequence, the yaw RMS error decreased from 3.76° to 3.21° over frames 10–28 and from 5.24° to 3.97° over frames 46–64, corresponding to reductions of 14.6% and 24.2%, respectively. Over frames 46–64, the translation RMS error also decreased by 33.5%. At frame 49, where the fixed template exhibited its largest rotation error, the absolute yaw error decreased from 11.13° to 6.39°, a reduction of 4.74° (42.6%). These results indicate improved overall tracking accuracy, with notable local gains under oblique viewing conditions.

### 3.4. Parameter Sensitivity Analysis

Parameter sensitivity was evaluated using the same 70-frame rotating sequence, initial CAD-derived template, and initial pose for all configurations. The baseline used $N_{\min}=5$, $d_{v}=5$ cm, and $\tau_{\theta }=45{}^{\circ}$. In this implementation, $d_{v}$ controlled both the temporal-support voxel grid and template-fusion downsampling; its variation therefore affected both observation selection and template resolution. Table 2 summarizes sequence-wide pose RMS errors, template size, and processing time.

Temporal support had the largest effect among the tested settings. Reducing $N_{\min}$ from 5 to 3 increased translation and rotation RMS errors from 4.37 cm and 3.32° to 15.58 cm and 14.17°, despite increasing the number of update frames from 66 to 68. With $N_{\min}=3$, the shorter temporal-support window admitted points displaced by pose-estimation drift into the template. These misregistered points distorted the reference geometry and reinforced subsequent registration errors, accounting for the larger tracking deviations in this run. Increasing $N_{\min}$ to 10 delayed the first update from frame 5 to frame 10 and reduced the update count to 61, but did not improve accuracy. These results favor the five-frame setting among the tested temporal thresholds for this sequence; they do not indicate that longer temporal support necessarily produces more accurate tracking.

Voxel size affected both pose accuracy and computational cost. A 2 cm voxel produced 29,282 final template points and required 264.14 ms per frame, while its translation and rotation RMS errors were both higher than those of the 5 cm baseline. A 10 cm voxel reduced the final template to 4155 points and the mean processing time to 63.62 ms, at the cost of increasing translation and rotation RMS errors to 4.97 cm and 3.61°. Thus, finer voxelization did not necessarily improve accuracy, whereas coarser voxelization reduced processing cost with an accuracy penalty in this sequence.

Tightening the incident-angle threshold from 45° to 30° gave slightly lower translation and rotation RMS errors, 4.25 cm and 3.28°, while retaining 66 update frames. Further tightening it to 15° reduced the update count to 62 and increased both errors relative to the baseline. The tested results therefore suggest a balance between restricting unfavorable viewing geometry and retaining observations for adaptation. These comparisons characterize parameter sensitivity for the tested rotating sequence and do not establish universally optimal settings.

## 4. Experimental Validation

Experiment I reproduced target rotation during an orbital inspection scenario, with viewpoint-dependent measurement loss. Experiment II used an approach sequence in which the fixed FoV covered an increasingly small, locally planar surface. Each ground-based sequence was processed separately with a fixed CAD-derived template, a fixed offline reconstructed template, and an online adaptive template. Registration residuals and attitude trajectories were compared to assess geometric agreement and tracking stability.

The three templates used in the ground-based experiments were constructed as follows:

- Fixed CAD-derived template: Template points were sampled from the CAD surface at a given resolution to represent the geometric prior. In both ground-based experiments, the CAD prior was a simplified geometric body without fine surface details.
- Online adaptive template: The method started from the same CAD-derived template as the fixed baseline and incorporated selected observations during tracking.
- Fixed offline reconstructed template: Multi-view scans from the same LiDAR were registered and fused before sequence processing. This independently reconstructed template provided a comparison based on measured geometry, including sensor-dependent surface details and missing–return patterns.

### 4.1. Experimental Hardware and Software Configuration

**Figure 5:**
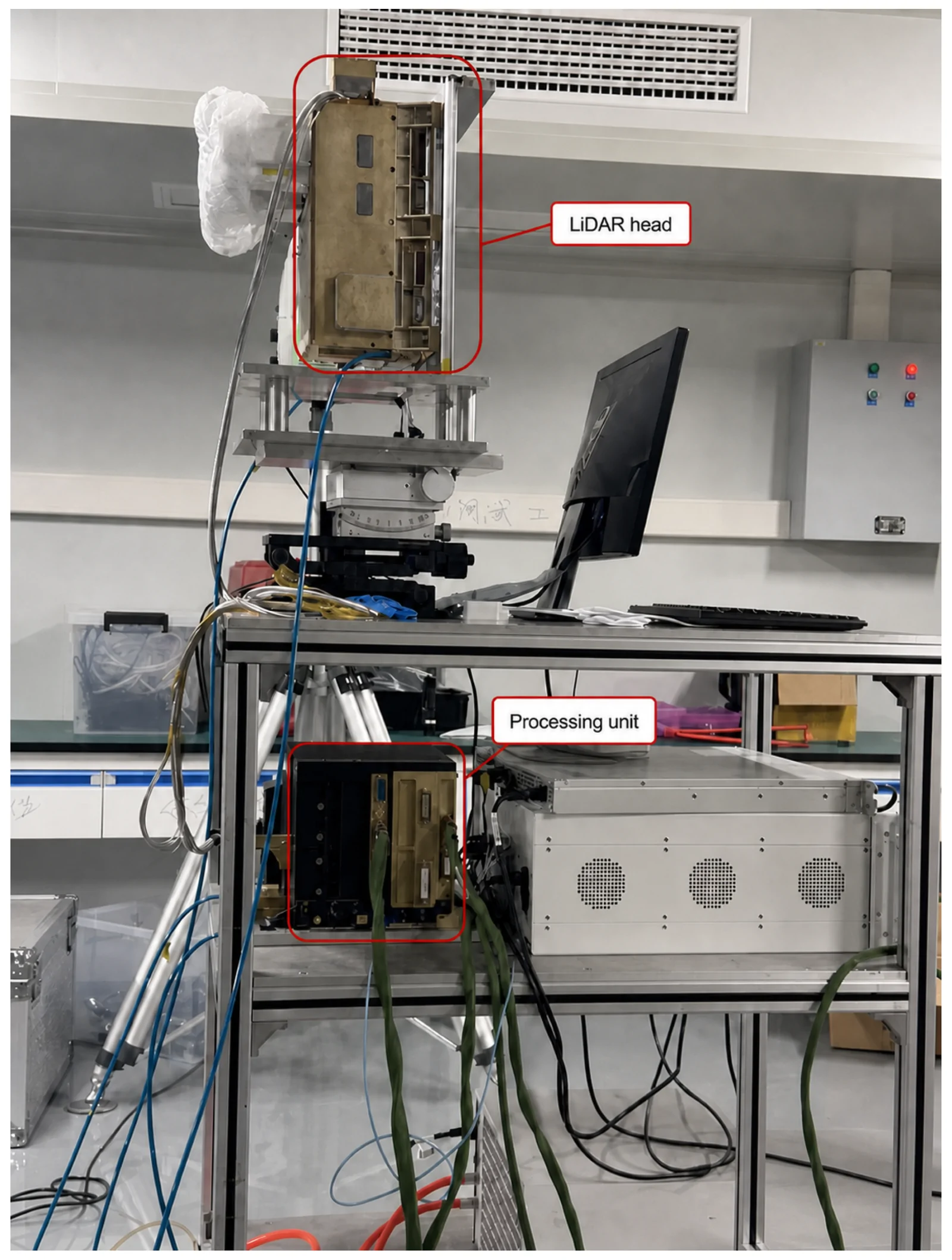
Photograph of the spaceborne 64-beam LiDAR sensor used in the experiments.

An in-house spaceborne 64-beam pulsed time-of-flight LiDAR developed at the Shanghai Institute of Technical Physics, Chinese Academy of Sciences, Shanghai, China, recorded the point clouds (Figure 5). The hardware specifications, baseline algorithm parameters, and computing platform are provided in Section 2.4 and Table 1. Both ground-based experiments used this configuration. Pose estimation and template adaptation were conducted offline on the recorded scans.

### 4.2. Experiment I: Pose Tracking in an Orbital Inspection Scenario

#### 4.2.1. Experimental Scenario

Experiment I used a scaled satellite with a central body and two solar panels to reproduce a rotational inspection scenario. The LiDAR stood approximately 30m from the target, which rotated about its principal axis at 5°/s. The evaluated sequence contains one frame per second, or approximately 5° of rotation between frames during the rotation phase. The range stayed fixed while the incident angles on the target surfaces changed.

**Figure 6:**
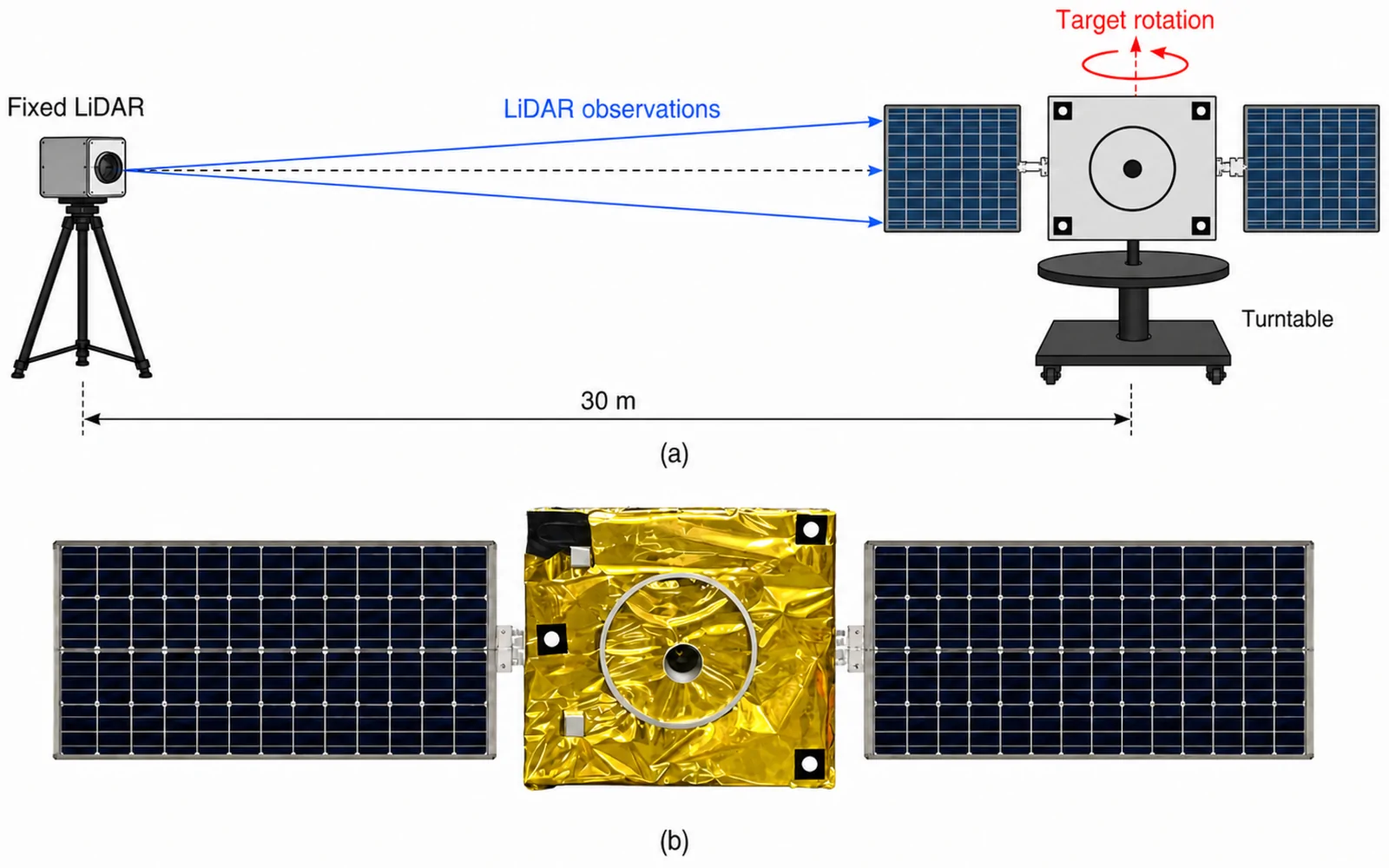
Experimental configuration of the orbital inspection scenario: (**a**) experimental setup; (**b**) scaled satellite model.

**Figure 7:**
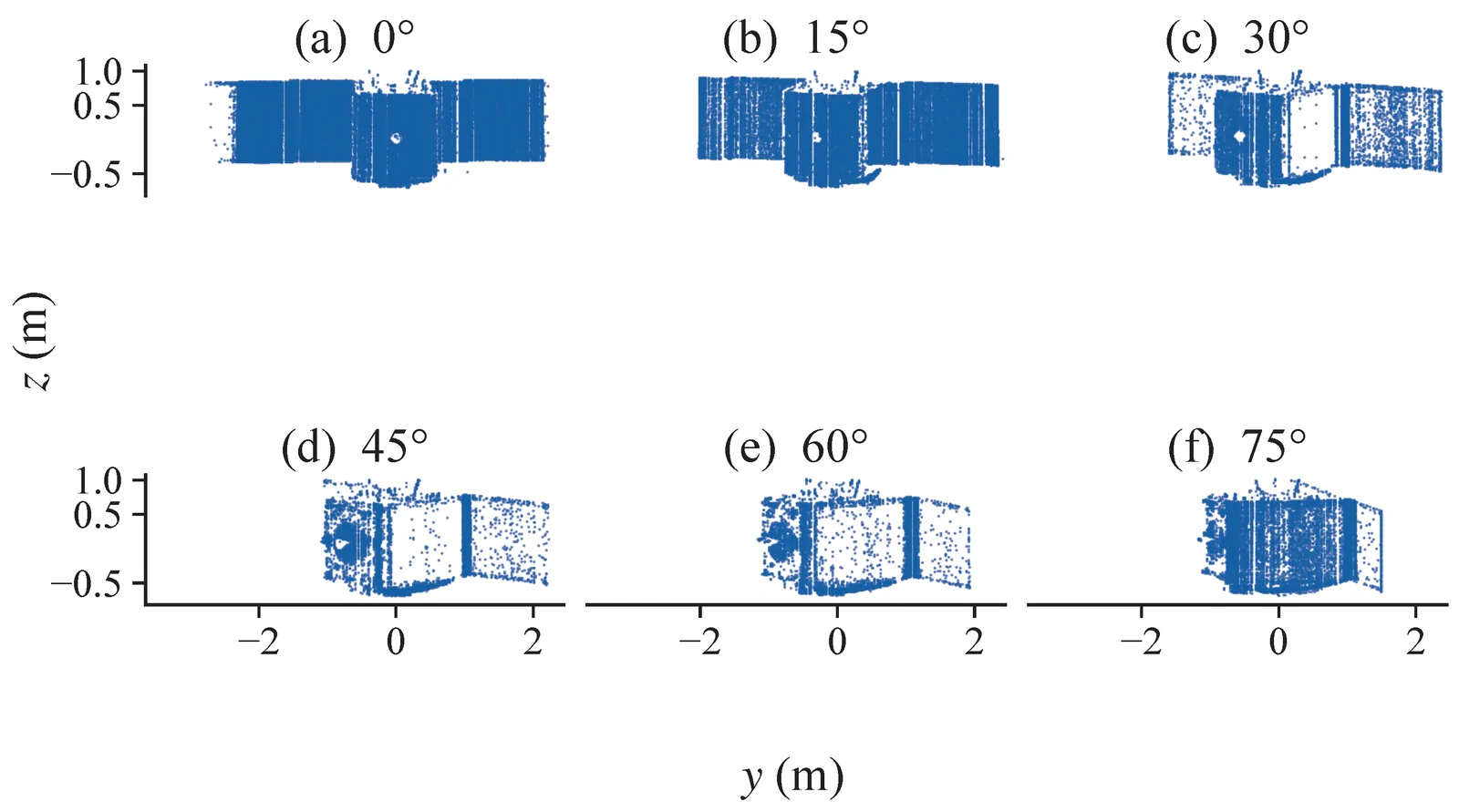
Representative LiDAR point clouds acquired at incident angles of 0°, 15°, 30°, 45°, 60°, and 75°.

Figure 6 shows the setup and satellite model. The central body was covered with multi-layer insulation (MLI), and the solar panels carried photovoltaic cell arrays. Both surfaces were highly reflective. At larger incident angles, the received echoes weakened and more returns were lost, leaving gaps on the body and panels. Figure 7 compares point clouds at incident angles from 0° to 75°. Point density and structural coverage decrease at larger angles, providing a test of tracking under viewpoint-dependent measurement loss.

#### 4.2.2. Target and Reference Templates

The fixed baselines used a simplified CAD-derived template and an independently reconstructed offline template. The simplified CAD model retained the main body’s dimensions but represented it as a smooth cuboid. Small attachments and surface details were omitted, while the main lower structure and side mounting plates were retained. The geometry was sampled and voxel-downsampled at 5cm. The fixed CAD baseline and online method started from this same approximate prior.

**Figure 8:**
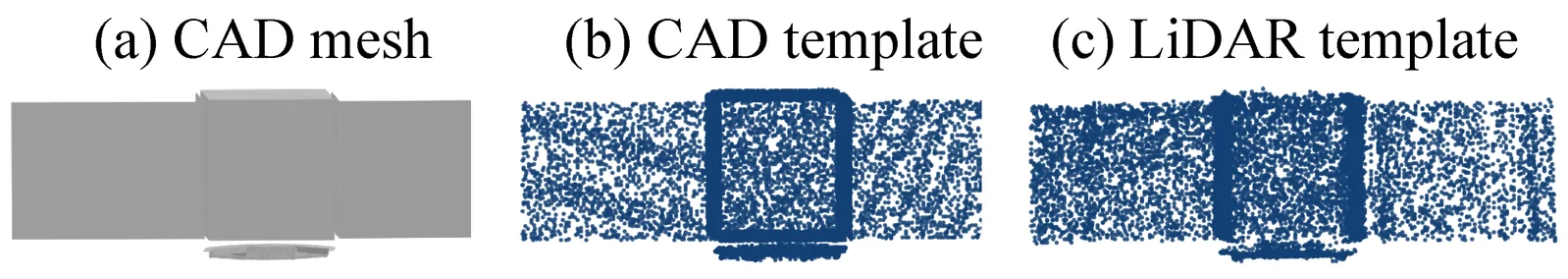
Target model and reference templates used in Experiment I: (**a**) simplified CAD mesh model of the scaled satellite target, (**b**) simplified CAD-derived point cloud template used for registration, and (**c**) offline reconstructed LiDAR template obtained from multi-view scanning.

The offline template was constructed by registering and fusing multi-view scans from the same LiDAR. Figure 8 shows the simplified CAD model and both fixed reference templates.

#### 4.2.3. Results and Discussion

**Figure 9:**
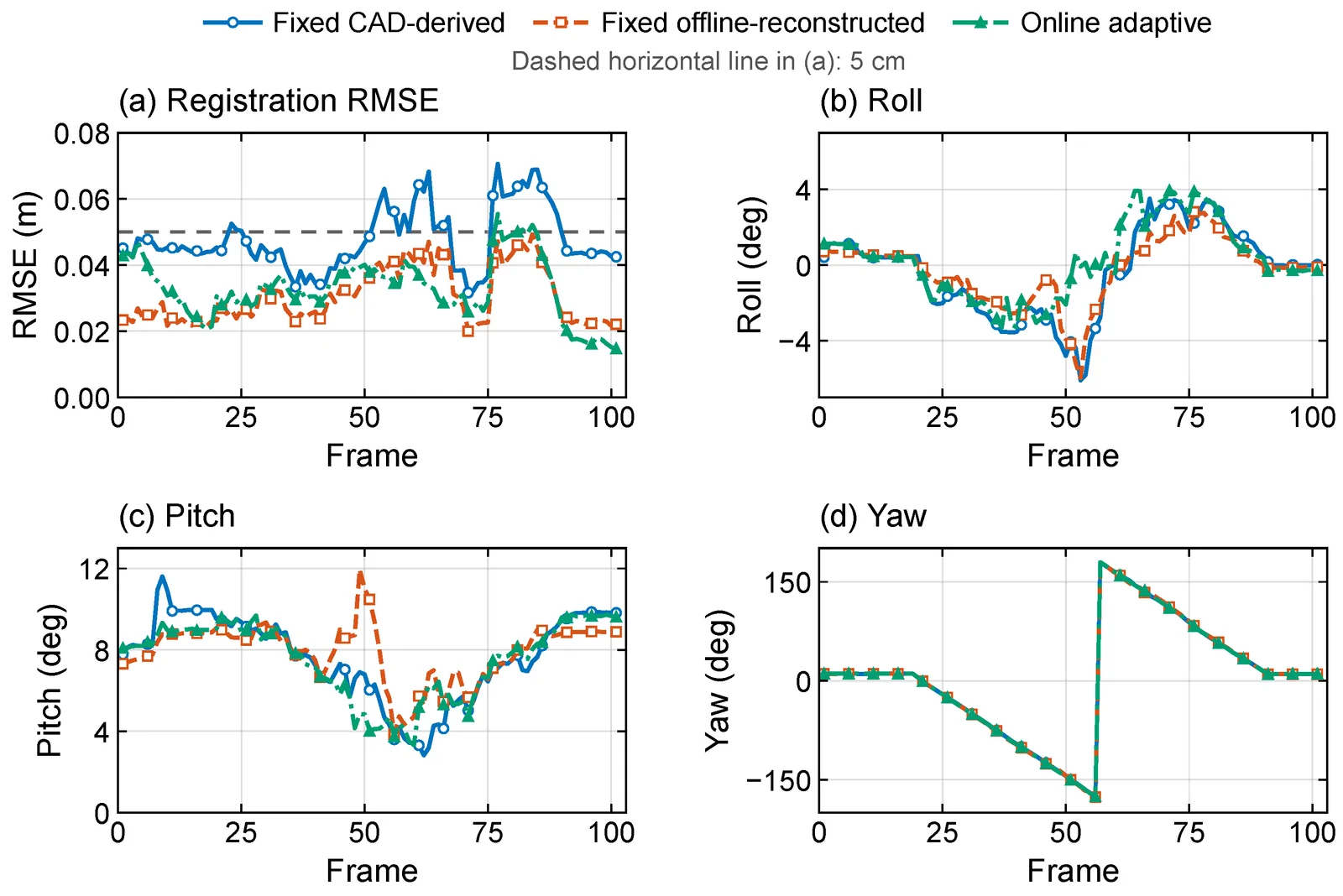
Experiment I results over 101 frames: (**a**) registration RMSE and (**b**–**d**) roll, pitch, and yaw extracted from the observation-to-template registration rotations. The dashed line in (**a**) denotes the 5cm (0.05m) tracking-quality threshold for switching from pose tracking to pose acquisition.

The three methods were compared over 101 LiDAR frames using registration RMSE and estimated attitude trajectories. Figure 9 shows the registration RMSEs and attitude trajectories. All three yaw trajectories covered approximately one target revolution, whereas the roll and pitch estimates differed locally. Mean RMSEs were 4.77cm for the fixed CAD-derived template, 3.05cm for the offline reconstructed template, and 3.29cm for the online adaptive template. Online adaptation reduced the mean RMSE by approximately 31% relative to the fixed CAD template; its mean was 0.24cm higher than that of the offline template. Under the 5cm tracking-quality threshold, the fixed CAD and online templates exceeded the threshold in 32 and four frames, respectively, while the offline template remained below it throughout. The four exceedances with the online template were isolated and non-consecutive, and therefore did not meet the persistence condition for switching back to pose acquisition. Compared with the fixed CAD template, online adaptation thus produced far fewer threshold exceedances and avoided tracking interruption during the target’s rotation, supporting improved tracking robustness.

Taken together, the RMSE and trajectory results suggest that online adaptation improved scan-to-template agreement relative to the fixed CAD template while preserving the overall rotational trend in this sequence. Here, RMSE measures geometric agreement with each method’s reference template rather than pose error relative to independent ground truth.

Computational performance was evaluated over the 101 frames of Experiment I. Each recorded scan contained an average of 12,191 points, ranging from 2682 to 24,434. Following denoising and voxel downsampling, an average of 3185 points per frame were retained for ICP registration, with a range of 1088–5434. The fixed CAD-derived and offline reconstructed templates contained 25,125 and 45,465 points, respectively, while the online adaptive template contained 22,662–45,000 points at registration.

Table 3 summarizes the total processing time per frame, including preprocessing, pose tracking, and template maintenance where applicable. The online adaptive method required 105.41±19.65ms per frame, with a maximum observed time of 147.92ms. All evaluated frames were processed within the 200ms interval corresponding to the nominal LiDAR frame rate of 5Hz, supporting computational feasibility on the tested laptop platform. Execution on an onboard embedded processor remains to be evaluated.

### 4.3. Experiment II: Pose Tracking in a Close-Range Observation Scenario

#### 4.3.1. Experimental Scenario

**Figure 10:**
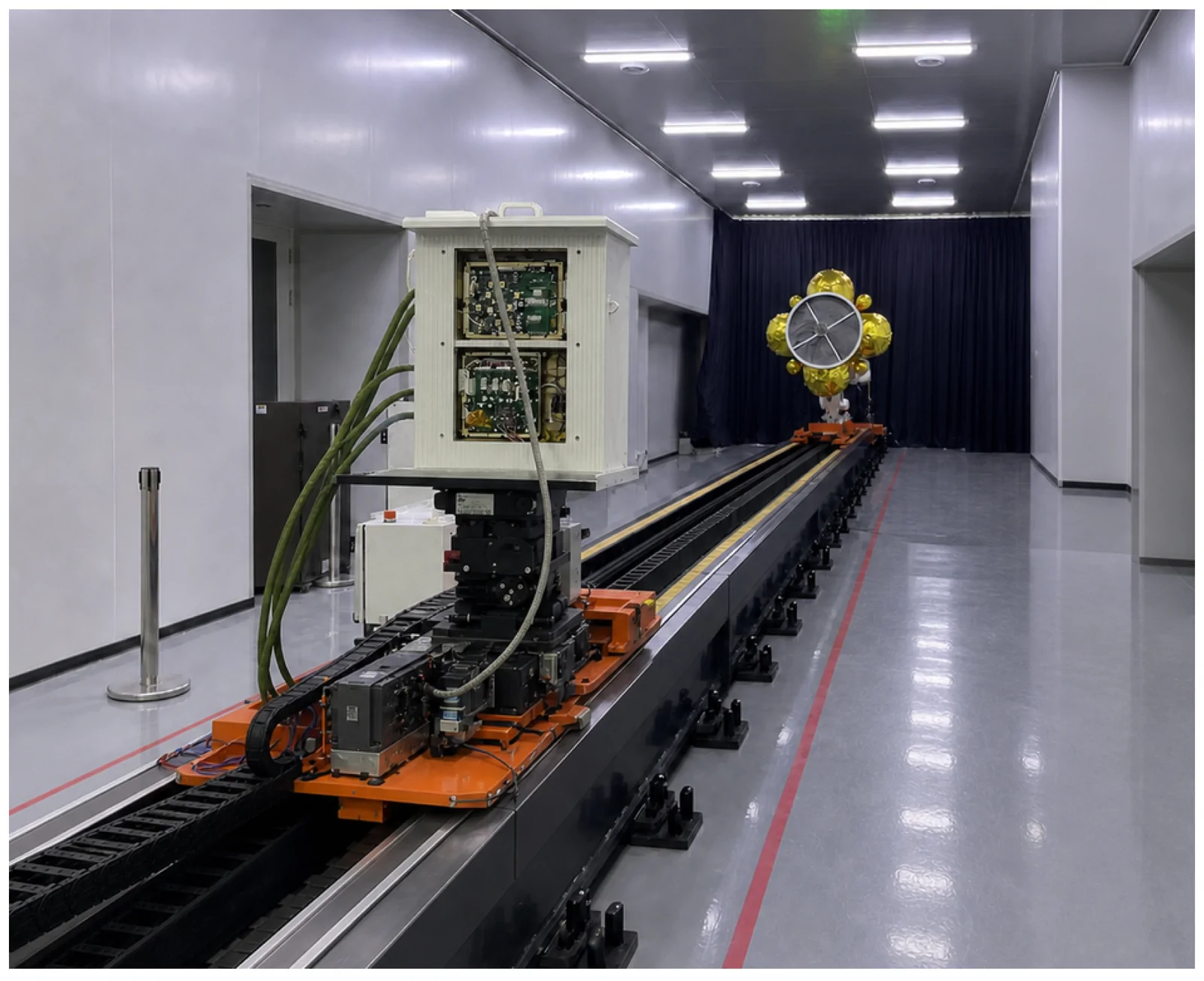
Experimental configuration of the close-range observation scenario.

Experiment II tested tracking as the LiDAR’s fixed FoV covered a progressively smaller part of the target. This examines a different observation condition from the changing incident angles in Experiment I. The target remained stationary while the LiDAR approached from 14m to 7.5m, recording 91 consecutive point clouds. Figure 10 shows the experimental configuration.

**Figure 11:**
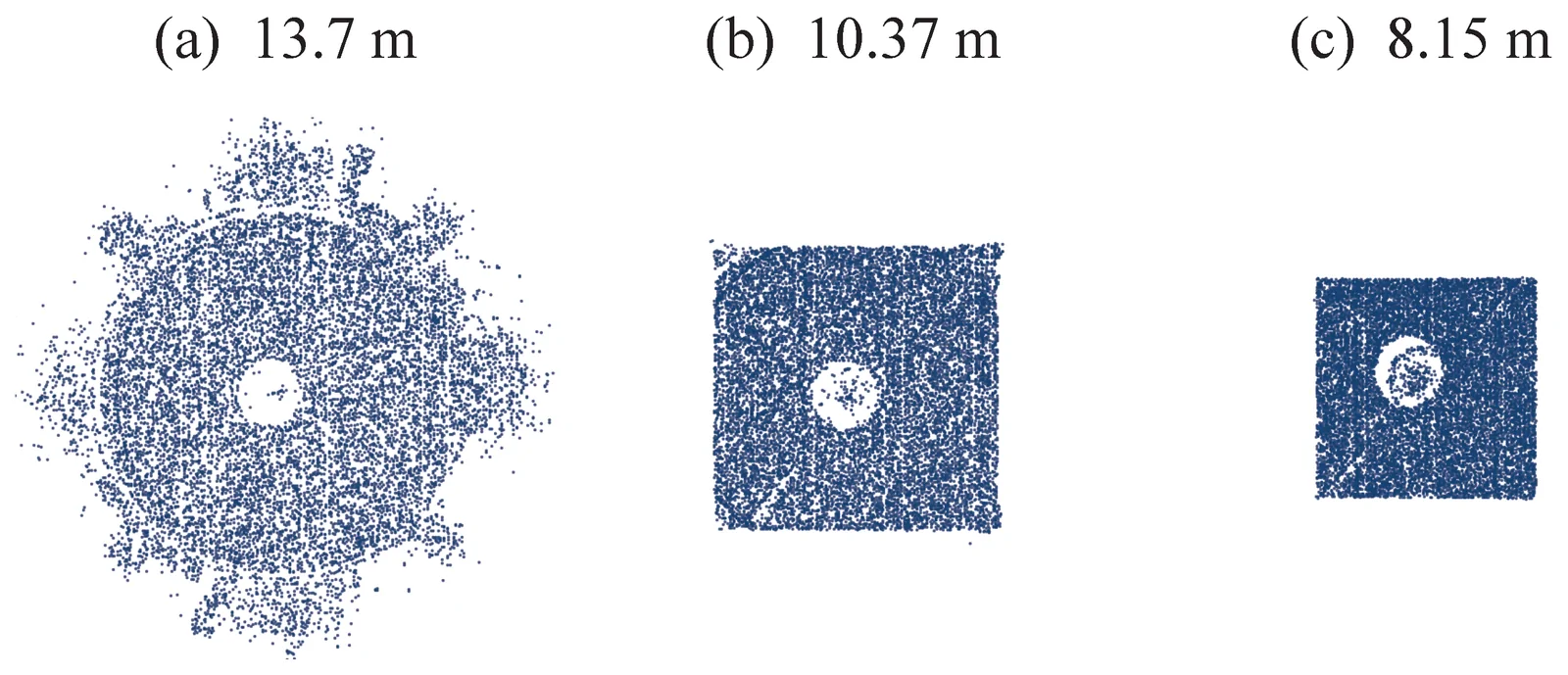
Representative point clouds acquired at observation distances of 13.7 m (**a**), 10.37 m (**b**), and 8.15 m (**c**).

As the range decreased, the observed surface changed from the full target to a small, approximately planar region. The loss of geometric diversity can make rotational registration more sensitive to measurement and alignment errors. Figure 11 shows the reduction in visible area at three observation distances. Only a local surface patch remains in view at the shortest distance shown.

#### 4.3.2. Target and Reference Templates

**Figure 12:**
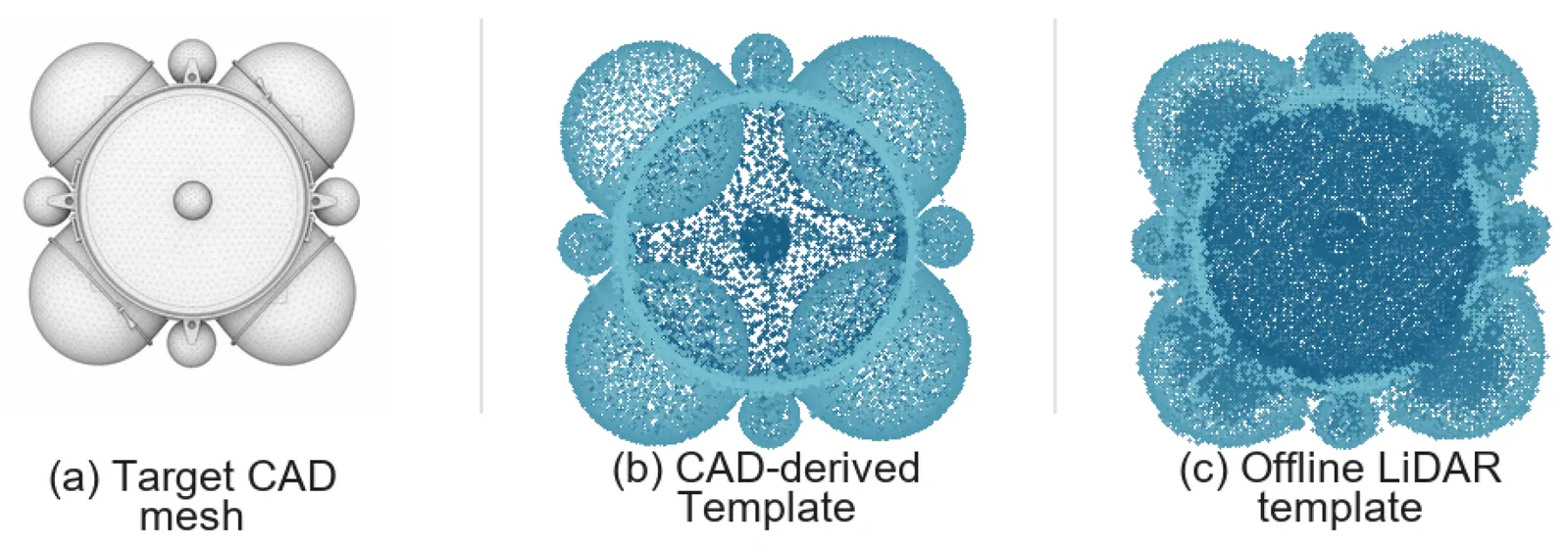
Target model and reference templates used in Experiment II: (**a**) CAD model, (**b**) CAD-derived template, and (**c**) offline reconstructed template.

Experiment II used a different spacecraft mock-up but the same two types of fixed reference templates: CAD-derived and offline reconstructed. The online method started from this target’s CAD-derived template and updated it during tracking. The CAD model and the corresponding reference templates are shown in Figure 12.

#### 4.3.3. Results and Discussion

**Figure 13:**
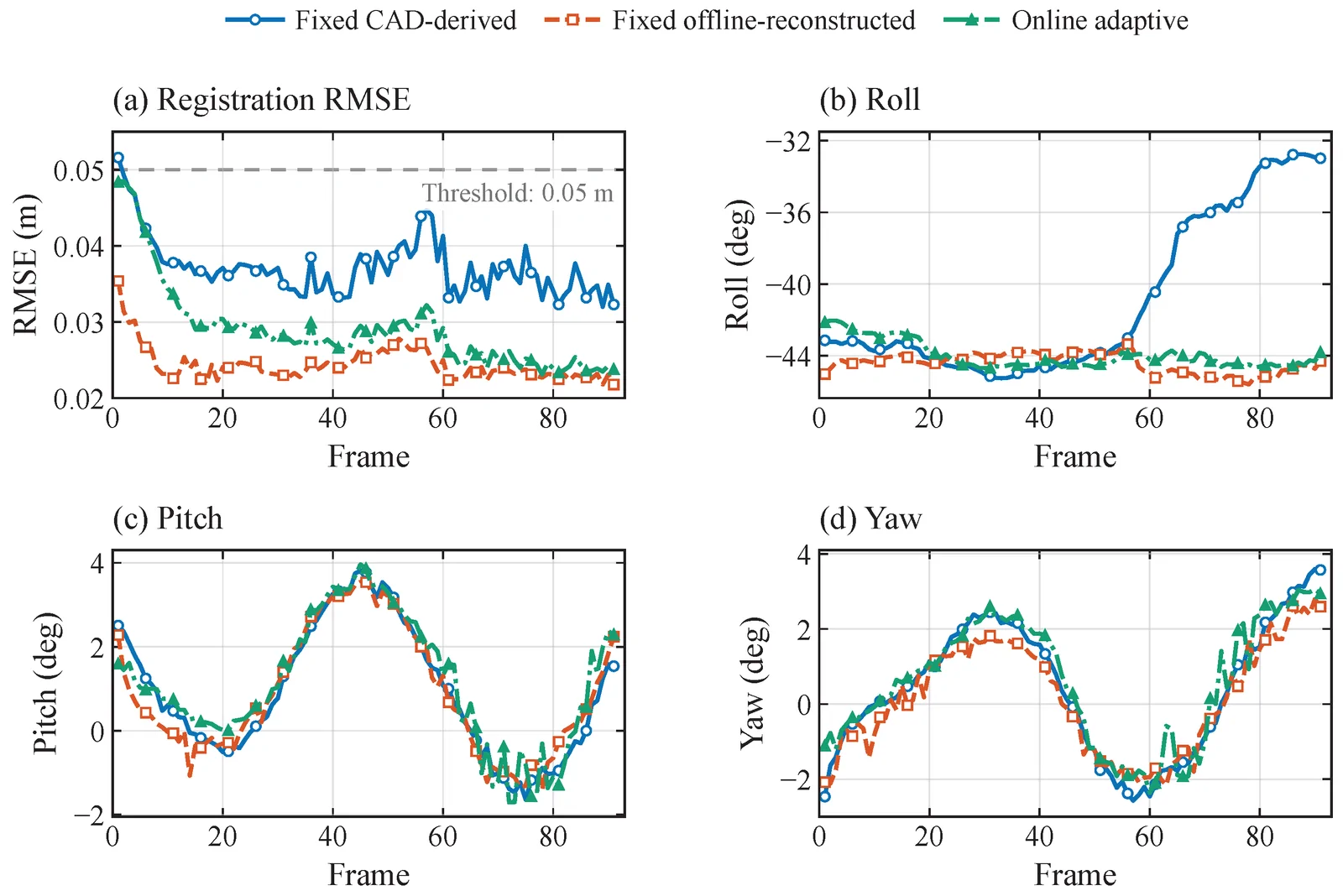
Comparison of pose-estimation results in Experiment II. (**a**) Registration RMSE and estimated (**b**) roll, (**c**) pitch, and (**d**) yaw trajectories over 91 LiDAR frames using the fixed CAD-derived template, fixed offline reconstructed template, and online adaptive template.

Figure 13 compares registration residuals and three-axis attitude estimates from the three methods over the 91-frame sequence. The offline reconstructed and online adaptive templates generally produced lower residuals than the fixed CAD-derived template, with values around 2–3cm for most frames. The online residual decreased during the early frames as observations were incorporated into the template. Pitch and yaw estimates were broadly consistent across the three methods. The fixed CAD roll estimate began to depart after approximately Frame 56 and drifted by more than 10°, while the offline reconstructed and online adaptive templates maintained comparatively stable roll estimates. During this drift, the fixed CAD residual remained around 3–4cm.

The target was stationary and the LiDAR motion was primarily translational, so the relative attitude was expected to remain approximately constant. As the range decreased, the fixed FoV contained only a small, nearly planar target region with few distinctive geometric features. Rotation about the local surface normal can then be partly accommodated by changes in nearest-neighbor correspondences, allowing small point-to-point residuals to persist despite an incorrect orientation. This weak rotational constraint is consistent with the roll drift observed for the fixed CAD template. The offline reconstructed and online adaptive templates included LiDAR-measured surface geometry, which provided the structure needed to maintain more stable roll estimates. The online method acquired this geometry during tracking, without a dedicated prior reconstruction. In this sequence, online adaptation maintained registration agreement and roll stability as the visible region became restricted, demonstrating its effectiveness under the tested close-range condition.

Computational performance was also evaluated over the 91 frames of Experiment II. Each input point cloud contained an average of 72,202 points, ranging from 52,618 to 77,414. Following denoising and voxel downsampling, an average of 2076 points per frame were retained for ICP registration, with a range of 1020–3070. The fixed CAD-derived and offline reconstructed templates contained 34,399 and 31,986 points, respectively, while the online adaptive template contained 31,802–40,851 points at registration.

Table 4 summarizes the total processing time per frame using the timing scope defined for Experiment I. The online adaptive method required 146.80±18.84ms per frame, with a maximum observed time of 179.97ms. All evaluated frames remained below the 200ms interval, supporting computational feasibility on the tested laptop platform for this close-range sequence.

## 5. Conclusions

This paper proposes an online reference template adaptation method for LiDAR-based pose tracking of non-cooperative targets, in which reliable observations selected by registration-quality, incident-angle, and temporal-support criteria are progressively fused into a CAD-derived template, requiring no dedicated prior multi-view reconstruction.

Two numerical simulations with known ground truth and two ground-based experiments evaluated the method under changing observation conditions. In the approaching simulation, online adaptation reduced translation and rotation RMS errors from 1.44 to 0.52 cm and from 1.10° to 0.40°, respectively, while limiting roll drift as the visible geometry became predominantly planar. The rotating simulation also showed lower sequence-wide pose errors, with notable improvements in the large-incidence-angle portions of the sequence.

In the ground-based rotational inspection experiment, online adaptation reduced the mean registration RMSE by approximately 31% relative to the fixed simplified CAD template (3.29cm vs. 4.77cm), approaching the offline reconstructed baseline (3.05cm), with threshold exceedances decreasing from 32 frames to four isolated, non-consecutive ones. In the close-range experiment, the online template maintained stable roll estimates as the visible region shrank to a locally planar surface, whereas the fixed CAD template drifted by more than 10° despite small residuals.

Together, the simulations provide quantitative evidence of pose accuracy against known ground truth, while the ground-based experiments support tracking stability under the tested sensing conditions. Small registration residuals alone do not guarantee accurate attitude estimates when visible geometry is limited. Long-term template drift remains to be evaluated, as persistent registration biases may accumulate through repeated updates despite geometric and temporal screening. Future work will investigate update suspension and rollback when tracking consistency deteriorates, and introduce independent pose ground truth into physical experiments.

## Figures and Tables

**Table 1:** LiDAR sensor specifications and template-adaptation parameters.

Parameter	Unit	Specification/Value
LiDAR sensor		
Measurement principle	–	Pulsed time-of-flight
Laser wavelength	nm	532
Laser configuration	–	1×64 linear laser array
Scanning mechanism	–	Two-dimensional, reciprocating and stepped
Angular resolution	deg	0.039×0.017
Single scanning strip	deg	20×2.5
Total field of view	deg	20×20
Measurement range	m	4–110
Range accuracy	cm	3
Imaging frame rate	Hz	5
ICP and online template adaptation		
Edge-feature weight, $w_{e}$	–	1.6
Planar-feature weight, $w_{p}$	–	0.4
Template-update residual gate, $\tau_{r}$	m	0.10
Point-to-template distance threshold, $\tau_{d}$	m	0.05
Incident-angle threshold, $\tau_{\theta }$	deg	45
Temporal-consistency count threshold, $N_{\min}$	frames	5
Template-update voxel size, $d_{v}$	m	0.05
Maximum ICP iterations	–	30
ICP translation convergence threshold	m	0.001
ICP rotation convergence threshold	deg	0.01
ICP residual-change convergence threshold	m	$10^{-4}$

**Table 2:** Parameter sensitivity on identical observations from the rotating scenario. Only the indicated parameter differed from the baseline. Translation and rotation RMS errors were calculated against ground truth over 70 frames. Time denotes the mean per-frame processing time for ICP and template maintenance, excluding observation generation, file I/O, and plotting.

Configuration	Translation	Rotation	Update	Final Template	Time
	RMS (cm)	RMS (°)	Frames	Points	(ms)
Baseline	4.37	3.32	66	15,129	163.85
$N_{\min}=3$	15.58	14.17	68	17,788	161.68
$N_{\min}=10$	4.70	3.98	61	13,384	168.73
$d_{v}=2$ cm	4.55	3.91	54	29,282	264.14
$d_{v}=10$ cm	4.97	3.61	66	4155	63.62
$\tau_{\theta }=15{}^{\circ}$	4.40	3.65	62	13,296	127.67
$\tau_{\theta }=30{}^{\circ}$	4.25	3.28	66	14,648	150.59

**Table 3:** Total per-frame processing time over the 101 frames of Experiment I. SD denotes standard deviation.

Method	Mean ± SD (ms)	95th Percentile (ms)	Maximum (ms)
Fixed CAD-derived template	38.43±6.89	48.99	61.10
Fixed offline reconstructed template	48.60±8.31	62.83	94.08
Online adaptive template	105.41±19.65	141.66	147.92

**Table 4:** Total per-frame processing time over the 91 frames of Experiment II. SD denotes standard deviation.

Method	Mean ± SD (ms)	95th Percentile (ms)	Maximum (ms)
Fixed CAD-derived template	68.85±3.41	72.79	77.77
Fixed offline reconstructed template	67.10±3.70	72.52	82.11
Online adaptive template	146.80±18.84	165.96	179.97

## Data Availability

The data supporting the findings of this study are available from the corresponding author upon reasonable request.
